# Maximizing Information Diffusion in the Cyber-physical Integrated Network †

**DOI:** 10.3390/s151128513

**Published:** 2015-11-11

**Authors:** Hongliang Lu, Shaohe Lv, Xianlong Jiao, Xiaodong Wang, Juan Liu

**Affiliations:** 1 National Key Laboratory of Parallel and Distributed Processing, National University of Defense Technology, Changsha 410073, China; E-Mails: shaohelv@nudt.edu.cn (S.L.); xdwang@nudt.edu.cn (X.W.); 2 Department of Computing, Hong Kong Polytechnic University, Hung Hom, Kowloon, Hong Kong, China; 3 College of Information System and Management, National University of Defense and Technology, Changsha 410073, China; E-Mail: xljiao@nudt.edu.cn; 4 School of Computer, National University of Defense Technology, Changsha 410073, China; E-Mail: juanl87@163.com

**Keywords:** cyber-physical network, information diffusion, relationship, dominating set, probabilistic links

## Abstract

Nowadays, our living environment has been embedded with smart objects, such as smart sensors, smart watches and smart phones. They make cyberspace and physical space integrated by their abundant abilities of sensing, communication and computation, forming a cyber-physical integrated network. In order to maximize information diffusion in such a network, a group of objects are selected as the forwarding points. To optimize the selection, a minimum connected dominating set (CDS) strategy is adopted. However, existing approaches focus on minimizing the size of the CDS, neglecting an important factor: the weight of links. In this paper, we propose a distributed maximizing the probability of information diffusion (DMPID) algorithm in the cyber-physical integrated network. Unlike previous approaches that only consider the size of CDS selection, DMPID also considers the information spread probability that depends on the weight of links. To weaken the effects of excessively-weighted links, we also present an optimization strategy that can properly balance the two factors. The results of extensive simulation show that DMPID can nearly double the information diffusion probability, while keeping a reasonable size of selection with low overhead in different distributed networks.

## 1. Introduction

**Figure 1:**
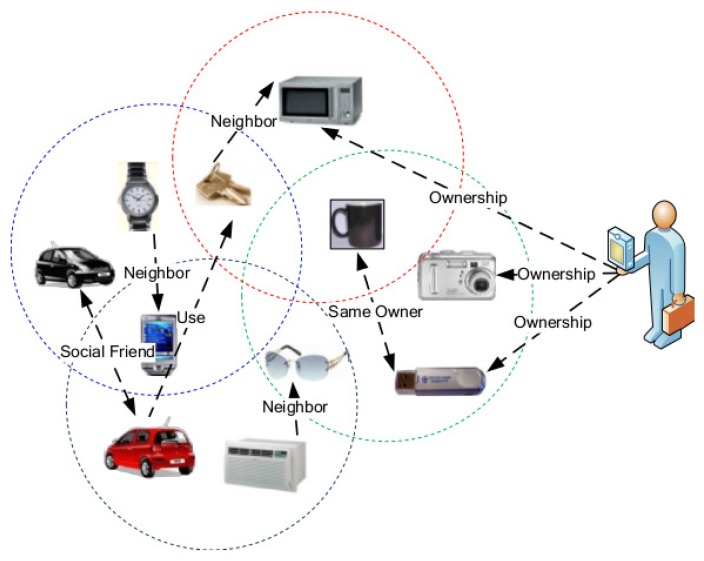
An example of a cyber-physical integrated network.

Advances in sensor networks have profoundly helped with integrating cyberspace with physical space. The integration makes mobile computing, smart sensing and smart controlling more sophisticated. It is expected that the integration of cyberspace and physical space will make our daily lives more convenient and efficient in the future. By connecting daily objects, such as televisions, cars, refrigerators, laptops, coffee cups, keys, *etc*., a heterogeneous network will be constructed. If the elements from cyberspace are added, the heterogeneous network will be correlated with the cyberspace, and a cyber-physical integrated network is formed. An example of such an integrated network is shown in Figure 1. Compared to the traditional Internet, that focuses on interconnecting computers, the cyber-physical integrated network connects both computers and various other types of physical objects. The cyber-physical integrated network is widely used in many fields, such as green computing, intelligent power service, smart cities and others. It is also regarded as an important data source and application area of big data technology.

Many applications are based on various kinds of relations between objects (as shown in Figure 1) in the cyber-physical integrated network. There are many dimensions to describe the relations, such as spatial dimension, temporal dimension, social dimension and others. Different relations are used to describe different attributes in corresponding dimensions. For example, the neighbor relation is used to describe the spatial relative position, which corresponds to the physical scenarios, e.g., a key resides near a laptop or a watch lies on a television, while co-owner relation is used to describe the social connection, which may refer to a car, a camera or a USB storage device belonging to a common owner. In other words, the relation is the key element for users to extract knowledge or to navigate among objects in the cyber-physical integrated network.

However, existing works related to the relations in the cyber-physical integrated network either focus on designing efficient algorithms to collect information or focus on the methods of extracting relations, few works studied how to use the relations to diffuse information. In previous works [1,2], the authors focused on collecting information from a collection of objects and using the collected information to infer relations between objects. They adopt a centralized strategy and collect information from a set of distributed objects to the centralized one. This introduces several unexpected problems, such as single points of failure, great difficulty in finding a strong enough server and serious security risks introduced by the centralized structure in the cyber-physical integrated network. Therefore, a distributed strategy is expected to satisfy the demands of applications in the cyber-physical integrated network.

In many interesting applications, several types of relations may be used, so that different types of information can be diffused in the cyber-physical integrated network. For example, in the scenario of accurate information flooding, certain types of information should be forwarded to a certain group of people, such as those engaged in advertisement, urgent temporary cooperation, emergency care, and so on. On the one hand, these types of applications have a high requirement of timeliness. On the other hand, the most typical characteristic of the relations in the cyber-physical integrated network is dynamic. We characterize the links between objects by the probability that they can communicate in a period of time, considering that most of the objects in the cyber-physical integrated network move all of the time. To meet the demands of information diffusion, we select the objects that can forward information to the target objects. The probabilistic features of the forward links guide us to consider maximizing the probability that information can be diffused in the network. Specifically, the problem becomes selecting a group of objects, so that the information diffusion probability is maximized.

In this paper, unlike previous approaches only focusing on minimizing the connected dominant set (CDS), we take the advantages of the CDS and consider the effects of light-weight links. Furthermore, we propose a distributed algorithm to select a group of objects to maximize the information diffusion possibility. In our design, the algorithm fully utilizes the neighbor information and optimizes the communication probability of the links. In the algorithm, additional intermediate objects are taken into consideration for enlarging the information diffusion opportunities. Extensive simulations are carried out to validate the effectiveness of the proposed algorithm. The results show that the proposed approach is effective and outperforms the existing approaches. In summary, this paper makes the following contributions.

- To the best of our knowledge, this is the first work to investigate the problem of probabilistic information diffusion in the cyber-physical integrated network.
- We proposed a distributed algorithm that can maximize the information diffusion probability.
- We conducted extensive simulations to validate the effectiveness of the proposed algorithm.

The rest of the paper is organized as follows: Section 2 describes the system model used in this paper. After that, the distributed object selection algorithm is illustrated in Section 3. The simulation results for the proposed algorithm are reported in Section 4. Section 5 reviews the related works, and finally, Section 6 concludes the paper.

## 2. System Model and Problem Formulation

The correlation of the cyber network and the physical network forms a more complex network. The integrated network is heterogeneous, both in the types of nodes and the diversity of links. A key characteristic of such an integrated network is its dynamics, which means that the nodes and the connections between nodes are changing all of the time.

### 2.1. Integrated Network Model

The cyber-physical integrated network has similar characteristics as sensor networks. Objects can communicate with each other when they lie nearby each other. We model the integrated network as a bidirectional graph G=(V,E), where *V* is the set of objects and *E* is the set of communication links. Keep in mind that the most important characteristic of the network is that several of the objects are moving all of the time. Thus, the constraints to set up a link between two objects have to be enhanced compared to the case of a sensor network.

In an integrated network, the links are setup if they satisfy the following constraints: given a time period from $t_{1}$ to $t_{2}$, ∀u,v∈V, there exists an edge E(u,v) between *u* and *v*, if and only if:

- *u* and *v* enter the data transmission range of each other during the time period.
- *u* and *v* communicate with each other during the time period.

In other words, the links are setup in a probabilistic way. This is because the property of the movement of the objects determined that the links between objects are not in a constant state, but change over time. Given a time period, we can not ensure that two objects will communicate with each other during the period, but we can estimate the probability that they communicate with each other based on the statistics of the history information. This strategy is used commonly in dealing with large networks. Thus, the weight of links can be represented by the probability that two objects communicate with each other during a certain period. For example, given a time period (12:00 to 12:30), $E_{u,v}=0.4$ means that during the time 12:00 to 12:30, the probability that *u* and *v* communicate with each other is 0.4.

Thus, the cyber-physical integrated network is time dependent. Given a time period, the integrated network is described by $G_{T}=\left(V,E\right)$, where $V=\{v_{1},v_{2},\cdots ,v_{n}\}$ corresponds to the objects, and *E* is the set of communication links between any pair of objects. Each $e_{ij}\in E$ has the weight of $P_{e_{ij}}$ that represents the probability of $v_{i}$ and $v_{j}$ communicating with each other.

**Figure 2:**
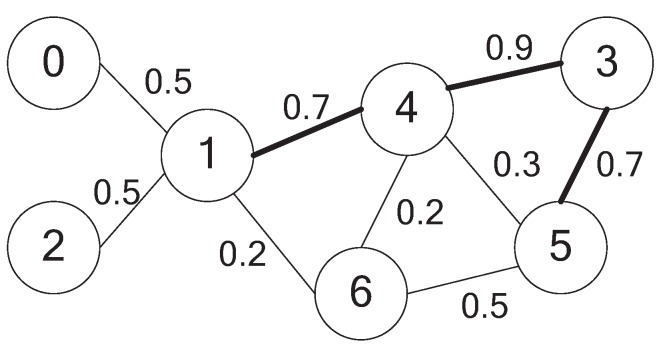
An example of an information diffusing probability network.

Under such a network model, to achieve the goal of diffusing information to a certain group of objects during a period of time in the cyber-physical integrated network, we have to select a group of objects that can maximize the information diffusion probability. The assumption is that the information is only forwarded between the selected objects, and the objects that are not selected can only receive information from the selected ones. However, the information diffusion probability is determined by the minimum weight in the network. For example, in the network shown in Figure 2, if we select the objects {1,4,3} as the forwarding objects, information can be forwarded between the selected objects under a probability of 0.7, but it is 0.2 when forwarding the information to all of the other nodes. This is because under such a selection, the information can only be forwarded to Object 6 by {1,4}; they both can accomplish this under a probability of 0.2. However, if we select the objects {1,4,3,5}, then the information can be diffused to all of the objects with a probability of 0.5, because in this case, Object 6 can receive information from 5 with a probability of 0.5, and the objects {0,2} can receive information from Node 1 with a probability of 0.5.

### 2.2. Problem Formulation

As described in Section 1, we want to maximize the information diffusion opportunity in the cyber-physical integrated network during a period of time. The critical task is to select the objects for information forwarding under two optimization goals: (1) to maximize the probability that other targeted objects receive the information; (2) to minimize the number of objects selected for information diffusing. The formal description is presented as follows:

Maximize the probability of information diffusion (MPID): Given a network $G_{T}=\left(V,E\right)$ and the distribution of the nodes, the MPID problem is to find a subset S⊂V, so that information can be forwarded to all of the target objects, and the optimization objective is:

- the size of *S* is minimized, *min*{|*S*|}.
- the probability for information diffusion is maximized, *max*{*Prob*(*S*)}

The MPID problem is a multi-objective optimization problem. The two objectives cannot be optimal at the same time. To make sure that information is forwarded to all of the target objects, we introduce the concept of the connected dominant set (CDS), where the selected objects should dominate the remaining ones in the network.

However, making a minimum selection that can maximize the information diffusion probability is an NP-hard problem. We give the proof in Theorem 1.

**Theorem 1.**  The MPID problem is NP-hard.

**Proof of Theorem 1.** Constructing a connected dominant set is proven to be NP-hard [3]. The MPID problem is similar to the problem of constructing a connected dominant set, as it also makes a selection so that the size of the selection is as small as possible and the probability is as large as possible. The final selection is dependent on the information diffusion probability represented by links. If we assume that all of the links in the network can diffuse information equally, then the network becomes the same as an unweighted graph. In this situation, the problem is exactly the same as that of constructing a connected dominant set in [3]. As a result, the problem is an NP-hard problem. ☐

## 3. Algorithm Design and Analysis

As described in the integrated network model, the cyber-physical integrated network is formulated as a bidirectional graph, and we design the algorithm based on the neighbor information. The prerequisite is that the information of the one-hop neighbors and the two-hop neighbors should be collected.

### 3.1. Distributed MPID Algorithm

In the cyber-physical integrated network, the problem of selecting a group of objects to diffuse information to all of the other objects has been demonstrated to be NP-hard. In such a network, traditional centralized methods are limited. Instead, distributed algorithms are promising to make the selection. Inspired by the distributed algorithms in wireless sensor network [4], we utilize the two-hop information to design our algorithm to select the objects.

For convenience, we define the dominant ability for both nodes and the set of nodes in the integrated network. Given a node *v* and its neighbor N(v), the dominant ability of *v* for its neighbor is defined as:

(1) DA(v)=α∗am(v)∗|LN(v)|+β∗CN(v)

In the equation, *α* and *β* are nonnegative coefficients to balance the effects of the links that have higher information diffusion probabilities, and the harm introduced by the links that have minor information diffusion probabilities, am(v), is the arithmetic mean of the probability of the links, *i.e.*,

(2) $$am\left(v\right)=mean\{P_{v,u}|u\in N\left(v\right)\}$$

The LN(v) is the set of links that a node can communicate with node *v* with a higher probability than am(v):

(3) $$LN\left(v\right)=\{e_{v,u}|P_{u,v}>am\left(v\right),u\in N\left(v\right)\}$$

The CN(v) is the difference between the arithmetic mean probability and the minimum probability of information diffusing from *v* to its neighbors.

(4) $$CN\left(v\right)=am\left(v\right)-min\{P_{v,u}|u\in N\left(v\right)\}$$

Intuitively, note that objects with more neighbors can forward information to their neighbors with a higher probability. Thus, such objects will play more important roles in a hop-by-hop communication network. To select these objects, as defined above, am(v) is respected to contribute for selecting the links that have higher information diffusing probability, LN(v) is respected to contribute for minimizing the number of objects to be selected and CN(v) is respected to contribute to the balancing of these two parameters.

Additionally, in some cases, a node and its neighbors can be dominated by another node. In such a case, the node can be pruned from the previous selection, so as to reduce the number of nodes that are selected to forward information. The ability for a node to dominate a set of other nodes is defined as:

(5) DAS({v},S)=α∗ams(v,S)∗|LS(v,S)|+β∗|CS(v,S)|

In Equation (5), $ams\left(v,S\right)=mean\left\{P_{v,u}|u\in S,\exists e_{v,u}\in E\right\}$, denoting the arithmetic average probability that information is forwarded from *v* to the objects in *S*. The $LS\left(v,S\right)=\{e_{v,u}|P_{v,u}>ams\left(v,S\right),u\in S\}$, is the set of links that a object can communicate with *v* in the set of *S* under a higher probability compared to ams(v,S). The $CS\left(v,S\right)=ams\left(v,S\right)-min\left\{P_{v,u}|u\in S,\exists e_{v,u}\in E\right\}$ is the difference between the arithmetic mean probability and the minimum probability of information diffusing from *v* to objects in *S*. Additionally, *α* and *β* are coefficients that are the same as the situation with the neighbor dominant ability, and they are network correlated.

In other cases, a node and its neighbor cannot be dominated by one other node, but can be dominated by multiple other nodes. In this case, even if the node is pruned from the selection, information can be forwarded to it or its neighbor. To depict this situation, we define the dominant ability of the set to set as:

(6) $$DAS\left(S_{1},S_{2}\right)=\alpha *ams\left(S_{1},S_{2}\right)*\left|LS\left(S_{1},S_{2}\right)\right|+\beta *CS\left(S_{1},S_{2}\right)$$

In Equation (6), $ams\left(S_{1},S_{2}\right)=mean\left\{max\left\{P_{u,v}|u\in S_{1}\right\}|v\in S_{2}\right\}$ denotes the arithmetic average probability that information is forwarded from objects in $S_{1}$ to the objects in $S_{2}$. For each object in $S_{1}$, the information is forwarded to $S_{2}$ through the links having maximum probabilities. The $LS\left(S_{1},S_{2}\right)=\left\{e_{u,v}|u\in S_{1},v\in S_{2},P_{e_{u,v}}>ams\left(S_{1},S_{2}\right)\right\}$ is the set of links that information can be forwarded from $S_{1}$ to $S_{2}$ under a higher probability than $ams(S_{1},S_{2})$. Additionally, the $CS\left(S_{1},S_{2}\right)=\left|ams\left(S_{1},S_{2}\right)-min\left\{max\left\{P_{u,v}|u\in S_{1}\right\}|v\in S_{2}\right\}\right|$ is the difference between the arithmetic mean probability and the minimum probability of information diffusing from objects in $S_{1}$ to objects in $S_{2}$.

Similar to the most distributed algorithm, we design the distributed MPID algorithm (DMPID) as a three-stage algorithm. In the first stage, the neighbor information and the roles (dominator or dominatee) are collected and determined for every object in the network. To be specific, each object collects its one-hop and two-hop neighbors’ information through package broadcasting. Based on the neighbor information, an initial role can be determined for each object in the network. For example, if two nodes *u* and *w* are both neighbors of node *v* and there is no direct communication link between them, *i.e.*, the link $e_{u,w}$ does not exist, the initial role of node *v* can be determined as the dominator, and it will be marked as blue.

The second stage of the DMPID algorithm is to reduce the number of dominators determined in the first stage. A dominator selected in the first stage will be pruned and marked as green by the following two criteria:

- a dominator and its neighbors can be dominated by one of its dominator neighbors, then the dominator can be pruned.
- a dominator and its neighbors can be dominated by a set of its dominator neighbors, then the dominator can be pruned.

By applying the two criteria mentioned above, the number of dominators is reduced in the second stage. However, in some cases, the reduction is harmful for maximizing the information diffusing probability. For example, in the network shown in Figure 2, Node 5 will be pruned from the dominator set after the second stage. However, if we retain it and introduce Node 3 as an additional forwarding node, the information diffusing probability would be much higher. Thus, the candidate objects to be pruned are rechecked by adding additional one-hop objects as forwarding nodes in the third stage. Taking the node *v*, which has to be pruned, as an example, if there exists a neighbor node *u* for *v*, then ∃u∈N(v) and u∈N(w), where *w* is a dominator, and $min\left\{P_{u,w},P_{u,v}\right\}>P_{w,v}$. We define the profit as the increment of the two information diffusing links compared to the original one, as Equation (7) shows.

(7) $$Pro\left(v,u|w\right)=\frac{min\left\{P_{u,w},P_{u,v}\right\}-P_{w,v}}{P_{w,v}}$$

If the profit is larger than a threshold *γ*, then the node *u* would be added as an additional forwarding node, and node *v* would be retained as dominator that forwards information in the process of information diffusion. The pseudocode of the DMPID algorithm is described as Algorithm 1.
**Algorithm 1** DMPID algorithm.**Require:** Each node knows information of its neighbor and two-hop neighbor.**Ensure:** A node knows the probabilities of the links to which it is attached.1:Initially, node *v* is marked as the dominatee.2:For node *v*, *u* and *w* are two of its neighbors3:**if**
*e_u,w_* does not exist **then**4:  the role of *v* is changed to be the dominator and marked as blue.5:**if** node *v* is a dominator (has been marked as blue) **then**6:  For each *v*’s neighbor *u*, that *DA*(*u*) ≥ *DA*(*v*)7:  **if** {*u*} can dominate *N*[*v*], and *DA*({*u*}, *N*[*v*]) ≥ *DA*({*v*}, *N*(*v*)) **then**8:   *v* is changed to be potential dominator, and marked as green9:  For nodes {*u*_1_, …, *u_k_*} ⊂ *N*(*v*), and *DA*(*u_i_*) ≥ *DA*(*v*), *i* = 1, …, *k*10:  **if** Set *S* = {*u*_1_, …, *u_k_*} can dominate *N*[*v*], and *DA*(*S*, *N*[*v*]) ≥ *DA*({*v*}, *N*(*v*)) **then**11:   *v* is changed to be the potential dominator and marked as green12:**if** node *v* is a potential dominator (has been marked as green) **then**13:  For each *v*’s neighbor *u* that is a dominatee14:  **if** a dominator *w* ∈ *N*(*v*), *w* ∈ *N*(*u*) and *Pro*(*v*, *u*|*w*) > *γ*
**then**15:   *v* is changed to be the dominator and marked as blue16:   *u* is changed to be the dominator and marked as blue


### 3.2. A Numerical Example

**Figure 3:**
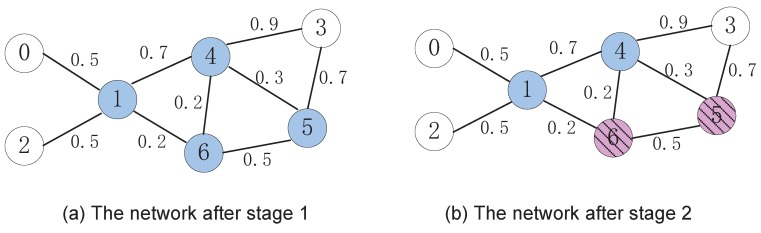
The state change of the network.

For clarity, a numerical example is presented to help with understanding the algorithm. The example network is shown in Figure 2. After the first stage of the algorithm, the role of each node is determined, and dominators have been marked as blue. The illustration is shown in Figure 3a. In the figure, the white nodes denote dominatees, and the blue ones are dominators chosen by the DMPID algorithm in the first stage. In the example network, after the first stage, a preliminary selection is made as S={1,4,5,6}. Take Node 1 as an example to describe the procedure of the first stage. When Node 1 checks its neighbor and finds that its two neighbors, 0 and 2, cannot directly communicate with each other, that is link $e_{0,2}$ does not exist, which can be determined by the two-hop neighbor information, Node 1 changes its role as the dominator and marks itself as blue.

After a node and all of its neighbors have determined their roles in the network, in the second stage, if the node is a dominator, then it will check the dominating abilities of its neighbor dominators. Take Node 5 as an example: it finds that its neighbor Node 4 is a dominator and the dominating ability DA(4)=1.05α+0.325β and DA(5)=0.5α+0.2β. Under the constraints of *α* and *β*, we can easily infer that DA(4)>DA(5). Besides, Node 5 and its neighbors can be dominated by Node 4. Since the neighbor set of Node 5 is N[5]={3,5,6} and the neighbor set of Node 4 is N(4)={1,3,5,6}, so N[5]⊂N(4). Due to Equation (5), DAS({4},N[5])=0.467α+0.267β, while DA(5)=0.5α+0.2β. As a result, with properly choosing the coefficients, DAS({4},N[5])>DA(5), *i.e.*, Node 5 and its neighbors can be better dominated by Node 4. Thus, Node 5 is pruned from the preliminary dominator set and marked as green. The result of the second stage is illustrated in Figure 3b.

**Figure 4:**
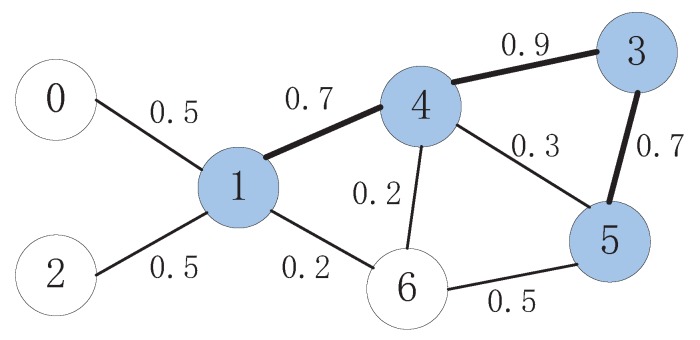
The final selection.

When a node and its neighbors have finished the pruning process, if the node is pruned from the preliminary dominator set, an additional check process is taken to retain it as a dominator, so that the information diffusing probability can be enlarged. This is the task that should be done in the third stage. Once again, take the network shown in Figure 2 as an example: Node 5 was pruned from the dominator set and marked as green after the second stage. By checking its neighbor, it can be found that if Node 3 is selected as the dominator, it is possible to retain it as the dominator. Due to Equation (7), the profit by adding Node 3 as an additional dominator would be Pro(5,3|4)=1.33. Thus, if γ<1.33, Node 5 would be retained as the dominator and marked as blue, and Node 3 would be added to the dominator set and marked as blue as well. Obviously, in this way, the number of nodes selected as the dominator in the final dominator set would be larger. However, if the advantage of the information diffusing probability introduced by the increment of the number of dominators is significant, it would be worthy to increase the selection. This will be discussed in detail in the next section. Finally, the DMPID algorithm will get the final selection shown in Figure 4.

### 3.3. Analysis

Distributed algorithms based on the neighbor and two-hop neighbor information have been proven to be valid and correct [4]. We take a similar strategy to design the DMPID algorithm, and every node in the network makes a decision based on the neighbor and two-hop neighbor information. From the perspective of every node, they are totally using the local information individually. Here, we briefly analyze the correctness, validness and complexity of the DMPID algorithm.

As stated in the network model that, given a time period, the communication links are generated based on the statistic of history information. During the given time period, objects related to the application are connected by the probability links and form a connected network. In the first stage of the DMPID algorithm, a node is selected as the dominator if there exist two neighbors that cannot communicate with each other directly. For example, *v* and *u* are two nodes in the network, but $e_{v,u}$ does not exist. Even if they cannot communicate with each other directly, information can be forwarded from *v* to *u* after several hops, since the network is assumed to be connected. The simplest situation is that the information is forwarded by an intermediate node *w* and, thus, *w* is selected as the dominator according to the algorithm. In this case, any node that can communicate directly with *w* and one out of *v* and *u* in the meantime will be selected as the dominator. In other cases, if the path from *v* to *u* is longer than two hops, then there exists a node on the path that is two hops from *v*. Thus, it is transformed to be the same as the situation ahead. Therefore, after the first stage of the algorithm, a connected dominator set will be selected. In the second and third stage of the algorithm, operations are taken with the precondition of guaranteeing the connectivity. As a result, the DMPID algorithm is shown to be valid.

After the first stage of the DMPID algorithm, a connected dominant set will be selected. Additionally, in the second stage, a pruning process is taken to reduce the number of nodes that have been selected. The criteria for pruning a node were stated previously, and the pruning process ensures that if a node *v* is pruned from the dominator set, information can still be forwarded to the node that depends on *v* that will forward the information. At the same time, the connectivity of other nodes in the dominator set is kept. Otherwise, the node cannot be pruned.

Besides, the DMPID algorithm is a heuristic algorithm that is based on neighbor and two-hop neighbor information. The selection of the dominator set is not optimal globally in terms of the information spread probability and the number of nodes to be selected. This is the same as most of the distributed algorithms [4]. In the DMPID algorithm, message exchange happens in the procedure of neighbor and two hops neighbor finding and dominator set pruning. The total message exchanged during the information diffusion procedure is comparable to the cases in most of the distributed algorithms [4]. In the cyber-physical integrated network, the computational complexity for each node relies on the number of its neighbor nodes. However, the number of neighbors for every node is bounded due to spatial and hardware restrictions in the cyber-physical integrated network. In short, the complexity of the algorithm is bounded.

## 4. Evaluation

The DMPID algorithm focuses on selecting a dominator set to make the information diffusing probability maximized. The most similar work we found is Lin *et al*. [4]. They tried to find a connected dominant set for a cognitive network. Additionally, the comparable parameters include the number of nodes in the final selection, the minimum weight of links in the final selected dominator set, the probability of information diffusing in our case and the network characteristics of the network formed by the nodes selected (such as diameter, density, and so on).

### 4.1. Evaluation Setup

The distribution of the network and the weight of the links affect the final selection heavily. The density of the nodes and the probability of the links between nodes are randomly generated in the simulation. We take the number of nodes as variable and observe the variation of the size of the selection and the information diffusing probability.

Firstly, we generate the network using a k-means clustering method to ensure the nodes’ distribution and density. We also keep the network as a connected network. Under this network model, the simulation is performed in two different scenarios, sparse networks and dense networks. In sparse networks, the links distribute with a probability of 0.3, while in dense networks, the distributed probability is 0.75. Besides, the probability for two objects’ communication is uniformly distributed. The coefficient for the dominant ability alpha is set as 0.7, and the beta is set as three. The simulation is run 50 times, and the average result is taken to reduce the influence of randomness. After that, we take the Watts-Strogatz model [5] to generate a small world network, where the neighborhood is restricted within two steps, the vertices of the lattice are connected and the rewiring probability is set to be 0.05. The other parameters are the same as the previous simulation.

### 4.2. The Results

The evaluation is performed to validate the algorithm’s correctness and efficiency in terms of the size of the selection, the maximum information diffusion probability and the characteristics of the network formed by the selected objects.

#### 4.2.1. The Size of the Selection

**Figure 5:**
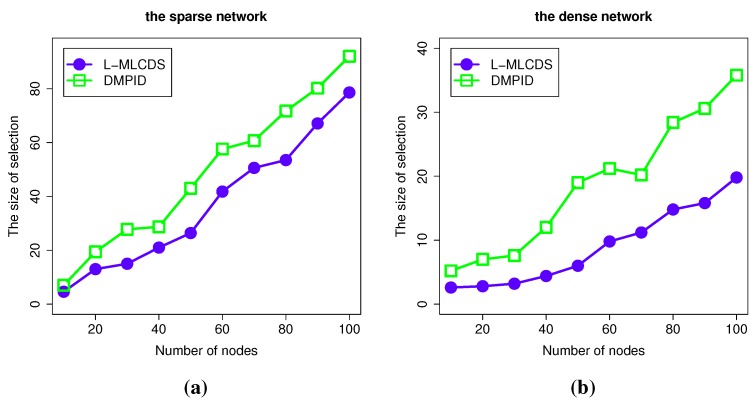
Simulation results for the size of the selection. The size of the selection in (**a**) the sparese network; (**b**) the dense network.

In the DMPID algorithm, the size of the selection is considered along with the information diffusing probability, which is the weight of the links. As stated in the algorithm design, in order to get a higher information diffusing probability, some additional nodes may be added to the selection, which makes the size of the selection a bit larger. In Figure 5, the size of the selection of the DMPID algorithm is a little bit larger. The reason is that some intermediate nodes are additionally selected to make the information spread probability higher. At the same time, in Figure 5, the size of the selection is comparable to the L-MLCDS [4] algorithm in the case of a sparse network. However, in the case of dense networks, the size of the selection is larger than that of the L-MLCDS algorithm. The reason for this is that when the density of the links is higher, there will be more chances that a higher information diffusing link exists. As a result, the intermediate nodes are chosen such that the size of the selection is enlarged. However, in Figure 5, we can easily find that the size of the selection is still within an acceptable level compared to the scale of the network.

#### 4.2.2. The Information Spread Probability

**Figure 6:**
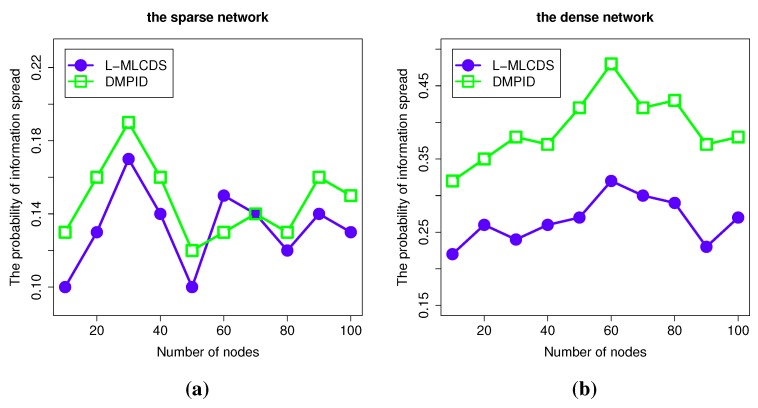
Simulation results for the information spread probability. The information spread probability in (**a**) the sparse network; (**b**) the dense network.

During any given time period, the information diffusing probability is bounded by the minimum probability link between the objects, which means that two objects have the lowest probability of communicating with each other compared to the other ones in the target group of objects. The DMPID algorithm tries to find the objects that can communicate at a higher probability and ensures that an object can communicate directly with at least one of the objects in the selection. In Figure 6, the DMPID algorithm can hardly get a higher information spread probability in a sparse network, because in a sparse network, there is little link information that can be forwarded to an object and there are few chances for an alternative link to be selected. Therefore, in most cases, the procedure of selecting an intermediate node fails. As a result, the communication links are kept the same as with L-MLCDS. However, in dense networks, there are more chances that intermediate objects are selected to enlarge the information diffusing probability. As a result, the information diffusion probability of the selection is higher in most cases. Additionally, in some cases, as shown in Figure 6, the results of the DMPID algorithm can be twice that compared to L-MLCDS in terms of the information diffusion probability.

#### 4.2.3. The Diameter of the Selection

**Figure 7:**
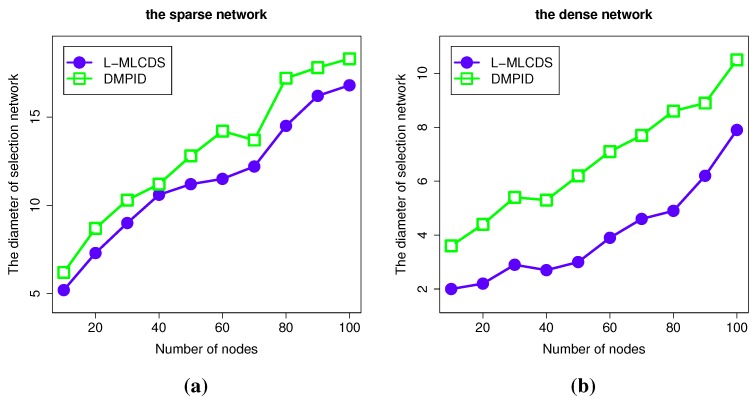
Simulation results for diameter of selection. The diameter of the selection in (**a**) the sparese network; (**b**) the dense network.

The diameter of a network is the farthest distance between pairs of nodes. We evaluate the diameter of the network formed by the nodes selected in the algorithm to show that the algorithm has a small effect on the communication efficiency. As shown in the left figure of Figure 7, in the sparse network, there is little probability that an additional node is added to enlarge the information diffusion probability, so the diameter of the selection is little changed compared to the L-MLCDS algorithm. In contrast, there are more chances to find an intermediate node to retain a dominator that is to be pruned from the preliminary selection. As a result, the algorithm has a relatively higher effect on the diameter. As shown in the right figure of Figure 7, in the dense network, the diameter is significantly increased. Compared to L-MLCDS, we only added an additional hop, so the maximum increment of the diameter is no more than four.

#### 4.2.4. The Density of the Network

**Figure 8:**
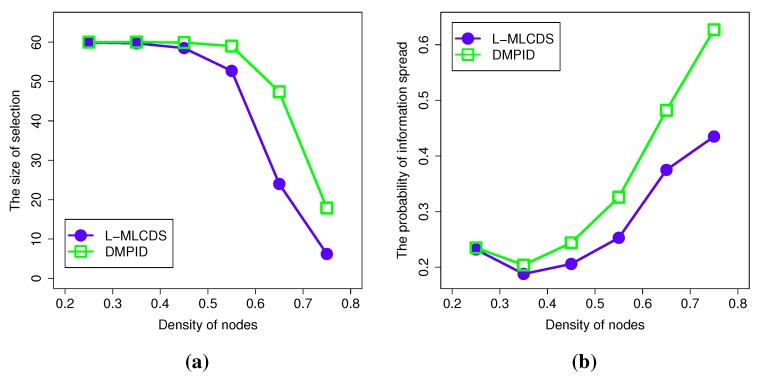
Simulation results for the density of the network. (**a**) The size of the selection; (**b**) The information spread probability.

The density of the links in the network affects the result significantly. We change the density of the links in the network and set the size of the network as 60 nodes. Then, we evaluate the size and the information diffusion probability of the network formed by the selected objects. As shown in the left figure of Figure 8, when the density of the links increases, the size of the selection decreases. This is because as the density of the links becomes larger and larger, a node can communicate with more and more nodes. As a result, the opportunity to choose fewer nodes to forward information to the remaining ones is increased. Since our algorithm is designed to add some additional nodes to get a higher information diffusion probability, the size of the selection generated by our algorithm is a little larger. This is reasonable, as the information diffusion probability of our algorithm is better, as shown in the right figure of Figure 8.

#### 4.2.5. The Results for a Network Generated by the Watts-Strogatz Model

**Figure 9:**
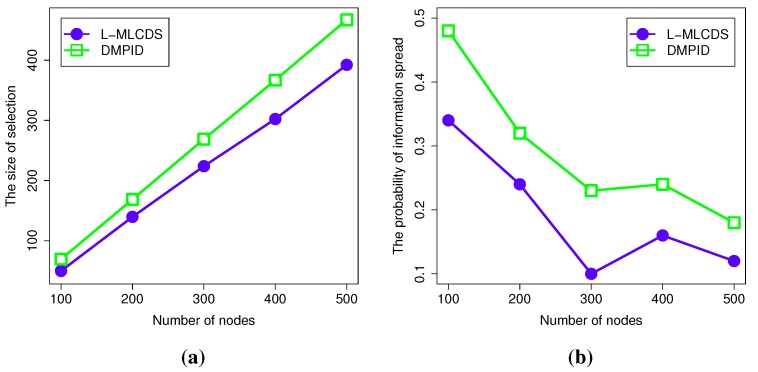
Simulation results for a small world network. (**a**) The size of the selection in small world network; (**b**) The information spread probability in the small world network.

Based on the Watts–Strogatz model, the network is generated as a connected network. Additionally, in such a network, a node *v* and its neighbor nodes *N*(*v*) have a small probability of being dominated by a node. At the same time, when the rewiring probability is very small, the probability that *v* and *N*(*v*) are dominated by a set of their neighbors is also very small. Here, we take two kinds of extreme cases as examples: the first case is the rewiring probability is one, and then, each pair of nodes in the network can communicate with each other directly; so in the first stage, no dominator will be selected. On the contrary, in the second case, the rewiring probability is set to be zero, then every node will be selected as the dominator, and none of them can be pruned in the second stage of the algorithm. Therefore, when we set the rewiring probability as 0.05, the final size of the selection is a little large. However, since there exist rewiring links, some of the nodes are still pruned. As shown in the left figure of Figure 9, the differences between two algorithms are slight. Furthermore, as the number of nodes grows, the gains are not changed obviously.

Besides, in the small world network, the information diffusing probability is closely related to the nodes selected as dominators. Take the two extreme cases as examples again: when the rewiring probability is set to be one, then any node can dominate other nodes in the network, so the information diffusing probability is the minimum probability that a node can communicate with the selected node. On the contrary, when the rewiring probability is set to be zero, then the information diffusing probability would be the minimum probability of a link in the network. As shown in the right figure of Figure 9, when such a minimum probability link is avoided, the information diffusing probability would be obviously enlarged. When the number of nodes increases, the information diffusing probability declines. The reason is that more nodes in the network lead to more communication links. This introduces the growth of the chances that small information diffusing links are generated. Thus, the final information diffusing probability is decreased.

## 5. Related Work

Information diffusion is a hot topic in social network research. The research includes modeling the information diffusion in specific forms of networks [6,7,8], speeding up the information diffusion in social networks to get a higher influence [9,10], exploring the diffusion means of some types of information in a social network and controlling or directing the information diffusion [11,12,13], *etc*. These pieces of works focus on the information diffusion in an online network, without considering the physical network.

There is rarely research on information diffusion in cyber-physical integrated networks. The work in [14,15] explored the impact of the speed and scale of information diffusion and knowledge creation by integrating the physical network, such as face-to-face communication, phone calls and the online social network (e.g., Facebook, Twitter, YouTube, *etc*.). Qian *et al*. [16] studied the information diffusion behavior of real-time information in an overlaying social-physical network. They assume that the physical network consists of many cliques and that information can spread quickly in the cliques. However, no matter how these cliques are formed, the information diffusion among cliques is not considered.

Selecting a dominator set in a wireless network is an important research topic. Many works have focused on minimizing the size of the selection and the efficiency of the selection process, where they assumed that the topology of the network is known ahead [17]. Selecting a dominator set in a wireless network in a distributed way can be divided into addition-based and pruning-based methods [18]. However, they consider the network links to be static [19]. The cyber-physical integrated network is a dynamic network, and the information diffusion problem in such a network must be handled considering that dynamism. The most similar work to ours is the work done by Lin *et al*. [4], where they studied the dynamic situation in a cognitive network.

## 6. Conclusions

This paper focuses on the information diffusion in the cyber-physical integrated network. We emphasize the dynamism of the network and the people-oriented information diffusion applications. In the distributed cyber-physical integrated network, the communication between objects is modeled as a probabilistic event based on the statistics of history information. To maximize the information diffusion probability to target objects, taking advantage of connected dominating set, we try to select a dominator set to diffuse the information. By considering the effects of small probability links that have had little attention paid, we present the DMPID algorithm. In the algorithm, the selection is made in a distributed manner using a pruning-based distribute algorithm design strategy. The results of the extensive simulation show that the DMPID algorithm performs well in different distributed networks. Future work includes the study of the information diffusion over the cyber-physical integrated network from the perspective of time migration and optimization for a large-scale small world network.
